# Hydroxocobalamin as Rescue Therapy in a Patient With Refractory Amlodipine-Induced Vasoplegia

**DOI:** 10.7759/cureus.38400

**Published:** 2023-05-01

**Authors:** Laith A Ayasa, Jehad Azar, Anas Odeh, Mohammed Ayyad, Sara Shbaita, Thabet Zidan, Noor Al-deen Awwad, Nagham M Kawa, Wafaa Awad

**Affiliations:** 1 Internal Medicine, Al-Quds University, Jerusalem, PSE; 2 Respiratory Institute, Cleveland Clinic, Cleveland, USA; 3 Faculty of Medicine, An Najah National University, Nablus, PSE; 4 Pediatrics, Al Makassed Hospital, Jerusalem, PSE

**Keywords:** amlodipine poisoning, hydroxocobalamin, vasopressors, distributive shock, vasoplegia

## Abstract

Vasoplegic syndrome is a type of distributive shock characterized by mean arterial pressure of less than 65 mmHg, with normal to high cardiac output and often refractory to fluid resuscitation, high doses of intravenous vasopressors, and inotropes. It is usually observed after cardiac and solid organ transplantation surgeries.

Here, we report a 56-year-old female patient who presented with a profound vasoplegia manifesting as lethargy and confusion in the setting of amlodipine toxicity. This case of severe vasoplegia was refractory to all conditional lines of medical management reported in the literature. The mainstay treatment modalities for vasoplegia include volume resuscitation, catecholamines, vasopressin, angiotensin II, and possibly methylene blue in unresponsive cases. Our patient was given hydroxocobalamin in favor of methylene blue, given the history of serotonin reuptake inhibitors use, which would have caused a life-threatening serotonin syndrome. Hydroxycobolamine resulted in a dramatic clinical recovery, suggesting its potentially significant role in refractory vasoplegia.

## Introduction

Vasoplegic syndrome is a form of distributive shock that is characterized by profound vasodilation, which leads to reduced systemic vascular resistance (SVR). This causes severe hypotension in the context of normal or increased cardiac output that is nonresponsive to fluid resuscitation [1]. Numerous factors, most notably elevated nitric oxide (NO) generation and immune dysregulation, are thought to be involved in the pathophysiology of vasoplegia. Vasoplegia is frequently observed in patients with sepsis, poisoning, burns, and trauma, as well as those undergoing cardiac surgery [2]. Interestingly, this condition has been treated with a variety of medications, including catecholamines, vasopressin, ascorbic acid, corticosteroids, and methylene blue [3]. Hydroxocobalamin, also known as vitamin B-12, has also been commonly used in the treatment of vasoplegia, through its effects on the NO system [4]. Refractory vasoplegia related to oral calcium channel blocker (CCB) treatment has rarely been reported in the medical literature. Herein, we report a case of amlodipine-induced refractory vasoplegia, which improved drastically following the administration of hydroxocobalamin. Given the patient’s drug history of selective serotonin reuptake inhibitors (SSRIs) use, methylene blue was not administered to avoid the development of serotonin syndrome. Given clinical recovery after hydroxocobalamin administration, the molecular absorbent recirculating system (MARS) was never needed in this case.

## Case presentation

A 56-year-old female patient was admitted to the intensive care unit (ICU) after presenting to the emergency department (ED) as a case of amlodipine overdose after a suicide attempt. She was found slumped over in her car and presented with drowsiness, fatigue, generalized weakness, nausea, and epigastric pain after ingesting 30 tablets of amlodipine 10 mg (300 mg total) in addition to a few tablets of buspirone. The patient has a medical history significant for psychiatric diseases including generalized anxiety disorder (GAD) and major depressive disorder (MDD), for which she was taking risperidone and buspirone. Additionally, there is a medical history of alcohol abuse and hypertension. Upon admission, vital signs revealed a blood pressure (BP) of 61/40 mmHg, a pulse in the range of 50 beats per minute, and oxygen saturation of 94%. Physical examination was otherwise unremarkable. Electrocardiography demonstrated sinus rhythm with a PR interval of 139 ms.

**Figure 1:**
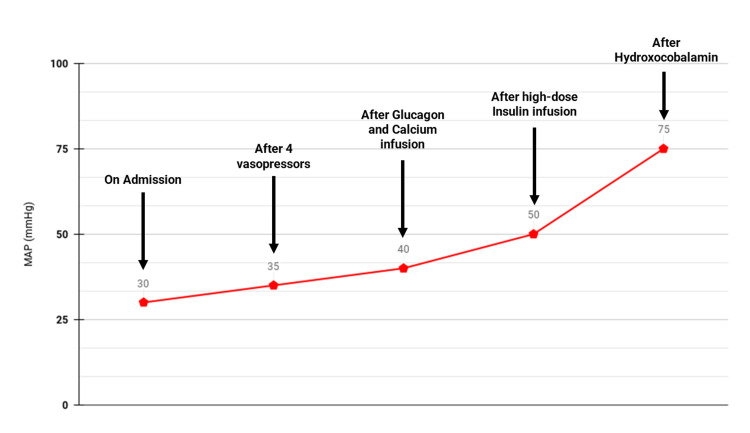
The hemodynamic changes (MAP) following the administration of vasopressor therapy This figure illustrates the hemodynamic changes (MAP) following the administration of vasopressor therapy, including glucagon, calcium, high-dose insulin infusion, and finally hydroxocobalamin. As per the figure, high-dose insulin and hydroxocobalamin proved successful in the management of our patient. MAP = mean arterial pressure

Within the first few hours in the ICU and as shown in Figure 1, the patient displayed signs and symptoms of severe, refractory hypotension as her mean arterial pressure was 30 mmHg following triple vasopressor hemodynamic support, including noradrenaline (120 mcg/kg/min), adrenaline (100 mcg/kg/min) and vasopressin (0.03 units/min). Bedside echocardiography revealed a hyperdynamic left ventricle, normal biventricular size, no regional wall motion abnormalities, and no pericardial effusion. Given the patient’s refractory vasodilatory shock state, phenylephrine was added (300 mcg/kg/min) alongside intravenous fluids (25 ml/kg/h) and an increased dose of vasopressin (0.06 units/min), but with minimal additional benefit as the vasoplegia persisted with a mean arterial pressure (MAP) of 40 mmHg. Her pulse was in the range of 70-90. She displayed worsening signs of tissue hypoperfusion with oliguria and severe lactic acidosis.

Bedside echocardiography showed no signs of cardiogenic shock with normal biventricular function. Consequently, the patient received glucagon and calcium infusions in an effort to circumvent the blockage of calcium channels by amlodipine, which didn’t result in significant improvement. Her MAP was 40 mmHg, pulse was in the range of 90-100 beats/minute, and oxygen saturation was 96% on room air.

The patient’s blood work was consistent with prolonged, severe hypotension and its complications revealing a severe, high-anion-gap metabolic acidosis; arterial pH 7.26 ( range 7.35-7.45), bicarbonate 11 mmol/L (range 22-26 mmol/L), anion gap 23.0 mEq/L (range 6-12 mEq/L), and lactate 12 mmol/L (range 0.5 to 2.2 mmol/L). Raised creatinine 1.87 mg/dL (range 0.5-1.1 mg/dL) and elevated blood urea nitrogen 23 mg/dL (range 7-20 mg/dL) confirmed acute kidney injury (AKI). Given the patient’s normal ventricular function, veno-arterial extracorporeal membrane oxygenation (VA-ECMO) was not considered as it would have resulted in a mixing cloud phenomenon (a watershed region). 

**Figure 2:**
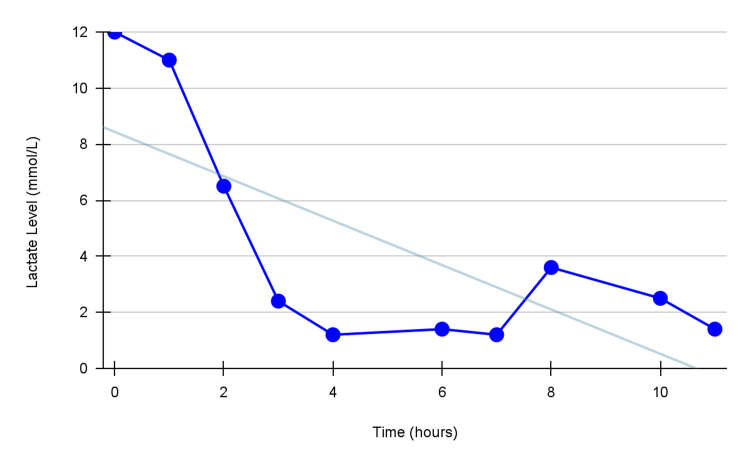
A line graph demonstrating the downtrend of lactate levels As the graph shows, there is a clear downtrend in lactate levels after the administration of hydroxocobalamin, suggesting that this treatment may be effective in improving outcomes in patients with refractory shock caused by amlodipine toxicity.

She was then given a high-dose insulin infusion with a 10% dextrose solution, which resulted in a slight elevation in the BP with a MAP of 50 mmHg. Following that, the patient was administered hydroxocobalamin 5 g, which significantly improved the BP 30 minutes later with a MAP of 75 mmHg and down-trending lactate levels as illustrated in Figure 2. Additionally, the creatinine level dropped to 0.99 mg/dL (range 0.5-1.1 mg/dL) and blood urea nitrogen level to 11 mg/dL (range 7-20 mg/dL). One hour later and due to improvement in her blood pressure and maintained stability, she was taken off all pressors.

Methylene blue is another potential medication that can be deemed useful in cases of amlodipine-induced refractory vasoplegia, but it was avoided in our case due to the risk of developing serotonin syndrome given her drug history including SSRIs. The last treatment modality that can be used to treat cases of severe, medication-resistant amlodipine-induced refractory vasoplegia is the molecular adsorbent recirculating system (MARS), as this system can clear protein-bound toxins. Amlodipine is highly protein-bound, and conventional modes of dialysis would not effectively clear the drug. MARS was not used in our patient given her hemodynamic improvement in response to high-dose insulin and hydroxocobalamin.

The patient displayed excellent clinical improvement during her observation period of 48 hours in the ICU. She was discharged to the internal medicine ward in a clinically stable condition and returned to her usual state of health a few days later.

## Discussion

Vasoplegia syndrome is a well-documented complication associated with both cardiac and solid organ transplantation surgeries. It affects 5-25% of patients following a cardiopulmonary bypass (CPB) [5], with an incidence as high as 30-50% in patients with predisposing risk factors [6]. It is characterized by a state of severe hypotension unresponsive to conservative management that includes fluid resuscitation, inotropes, and vasopressors, due to significant pathological vasodilation, leading to a profound reduction of the SVR with preserved cardiac output. There is no consensus on a numerical definition of vasoplegia, but it's most frequently described as exhibiting a cardiac index of 2.2 L/min/m^2^, either normal or elevated, accompanied by difficulty maintaining a MAP of 60 mmHg due to an SVR of less than 800 dynes*sec/cm^5^, despite receiving high doses of vasopressors and inotropes (equivalent to 0.5 mg/kg/min of norepinephrine) [7].

Vasoplegia syndrome has a complex pathophysiology that remains poorly understood. Emerging theories suggest that it might be related to reduced arginine vasopressin plasma levels, increased nitric oxide (NO) generation [8], increased adenosine release [9], and immune-mediated cytokine release [10]. 

**Figure 3:**
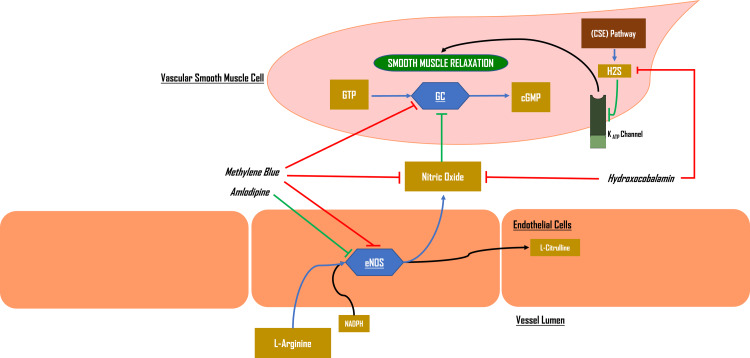
The nitric oxide pathway Endothelial nitric oxide synthase (eNOS) metabolizes L-arginine into nitric oxide (NO), which in turn acts on guanylate cyclase (GC) to induce vascular smooth muscle relaxation. (Ayasa L. (2023). Hydroxocobalamin as Rescue Therapy in a Patient With Refractory Amlodipine-Induced Vasoplegia. Al-Quds University)

Nitric oxide (NO) is a smooth muscle relaxing factor that is generated from L-arginine by the action of endothelial nitric oxide synthase (eNOS) [11] and has an important role in the pathophysiology of vasoplegia [12]. NO exerts its vasodilatory effects through the activation of soluble guanylate cyclase, which catalyzes the conversion of GTP to cyclic GMP leading to myosin phosphorylase activation (Figure 3) [13]. Another vasodilatory system involved in vasoplegia is the hydrogen sulfide (H2S)/cystathionine-y-lyase (CSE) pathway, in which the diffusion of hydrogen sulfide to smooth muscle cells causes the activation of potassium channels at high concentrations; which subsequently results in the hyperpolarization of the cell membrane and closure of voltage-gated calcium channels, leading to decreased Ca2+ entry and vasodilation [14].

During surgery, tissue injury and hypoperfusion can activate immune cells, such as macrophages and monocytes, leading to the release of pro-inflammatory cytokines such as tumor necrosis factor α (TNF-α), interleukin-1β (IL-1β), and IL-6. These cytokines decrease the responsiveness of vascular smooth muscle cells to vasoconstrictors, causing vasodilation and decreased SVR. The activation of the complement system can also contribute to vasoplegia by releasing vasoactive peptides, such as bradykinin and C5a, leading to vasodilation and increased vascular permeability [15].

However, the literature is lacking in regard to cases similar to ours, that is toxicity-induced vasoplegia. Amlodipine overdose, for example, has a significant impact on SVR due to the increased release of NO through the activation of eNOS [16], and direct blockage of the voltage-dependent L-type calcium channel preventing the initial influx of calcium. Consequently, actin and myosin interactions will not occur effectively, resulting in diminished vascular smooth muscle contractility and subsequent vasodilation and shock [17]. In general, under aerobic conditions, myocytes require and utilize glucose as their main source of energy instead of free fatty acids. The glucose, however, will not be used adequately due to amlodipine-induced blockage of L-type calcium channels in pancreatic beta cells that decreases insulin release impairing glucose utilization [18]. Due to its protracted half-life (between 30 and 60 hours) and delayed commencement of action, amlodipine concentration in the plasma can be elevated for days, which increases the concern for developing fatal toxicity [19].

In general, CCB-induced vasoplegia is commonly reported as a complication of subarachnoid hemorrhage treatment rather than intentional toxicity. A case series of four patients by Bele S et al. was the first to report refractory vasoplegia resulting from nimodipine administration, which was successfully resolved by administering methylene blue [20]. Similarly, Clifford KM et al. were pioneers in describing the use of hydroxocobalamin for CCB-induced vasoplegia, which led to a significant increase in MAP within minutes of medication administration [21]. Cooper TM et al. also reported a case of nimodipine-induced vasoplegia that was unresponsive to intravenous fluid boluses, phenylephrine, and norepinephrine. Although methylene blue was initially administered, it did not have any beneficial effects due to medication extravasation. However, the patient was successfully weaned off all vasopressors within 15 minutes of administering hydroxocobalamin as a rescue therapy [22].

The clinical presentation of vasoplegia usually involves altered mental status, lack of alertness, confusion, and rapid progression to loss of consciousness. On physical exam, patients will have hypotension, tachycardia, tachypnea, warm extremities with bounding pulses, and skin that is first warm and later becomes cold and clammy [23]. If possible, a thorough and focused history should be obtained from the patient. This is frequently not feasible, thus information should instead be gathered from family members or other witnesses of the inciting event, as most patients will have a clear surgical or pharmacological history. It is important to note that the clinical presentation of vasoplegia may overlap with other conditions such as sepsis, anaphylaxis, or cardiogenic shock. Therefore, a thorough clinical evaluation is essential to establish the underlying cause of vasoplegia and initiate the appropriate treatment.

Although vasoplegia is a clinical diagnosis, imaging can play an important role in the diagnosis and management of vasoplegia. The choice of imaging modalities may depend on the underlying cause of vasoplegia and the organs involved. Echocardiography can provide valuable information on the heart's structure and function, which is important to rule out cardiogenic shock. Chest X-ray may be useful in evaluating the lungs and detecting any abnormalities that may contribute to vasoplegia, such as pneumonia or pulmonary edema [24]. Invasive procedures are not usually required for diagnosing vasoplegia but may be necessary in some cases to determine the underlying cause such as right heart catheterization or echocardiography, for ruling out cardiac dysfunction [25]. The need for these procedures should be assessed on a case-by-case basis while considering the patient's clinical status and potential risks and benefits.

There is presently no "standard of care" treatment protocol for amlodipine-induced vasoplegia. Recent advancements in pharmacotherapy have opened up treatment choices for the majority of patients. However, caution should be exercised, taking into account each agent's unique adverse effects. In addition to volume resuscitation, high-dose insulin, angiotensin II, and traditional vasoconstrictors, vasopressin, terlipressin, methylene blue, hydroxocobalamin, and MARS have been reported in the literature as possible effective therapeutic modalities [26,27]. Although many agents were described for refractory vasoplegia, methylene blue and hydroxocobalamin were the most reported and useful ones [28]. Their early use as a rescue therapy diminished the need for other vasopressors and reduced overall mortality [29,30]. Due to the lack of standardized treatment guidelines, Ortoleva JP and Cobey FC have suggested an algorithm based on medication side effects and limitations considering the risk of concomitant serotonin syndrome or glucose-6-phosphate dehydrogenase (G6PD) deficiency [7].

The mechanism of action for methylene blue is through its inhibitory effects on the synthesis of cyclic Guanosine 3',5'-cyclic monophosphate (cGMP), eNOS, and NO. It also prevents vascular smooth muscle relaxation. Moreover, by interacting with endothelial muscarinic (M3) receptors, it inhibits cholinesterase and helps adjust the hemodynamic response by lowering the baseline NO release [31]. Methylene blue also inhibits the action of monoamine oxidase A, the enzyme responsible for breaking down serotonin in the brain [32]. This specific interaction is responsible for the life-threatening complication known as serotonin syndrome in many reported cases [33]. Methylene blue infusion can cause mild to severe, dose-dependent adverse effects such as nausea, vomiting, methemoglobinemia, hyperbilirubinemia, chest discomfort, and pulse oximetry interference [34].

Hydroxocobalamin is an established treatment modality for acute cyanide toxicity, which has been used as an off-label rescue treatment for vasoplegia. In contrast to methylene blue, it has not been linked to an elevated risk of serotonin syndrome and is not known to have any direct effects on the serotonergic system. In a patient who had hypotension while receiving cardiopulmonary bypass, Roderique et al. reported the use of hydroxocobalamin for treating catecholamine-resistant vasoplegic syndrome. Because their patient was chronically using citalopram, a particular serotonin reuptake inhibitor, and was therefore at risk of developing serotonin syndrome, the authors decided not to administer methylene blue and instead use hydroxocobalamin. Subsequently, the MAP increased from 20 mmHg to 80 mmHg, with a successful reduction in the required doses of vasopressors [35].

Less is known about the mechanism of hydroxocobalamin's hemodynamic effects. Studies involving human and animal subjects have consistently shown that ingesting hydroxocobalamin was associated with an increase in blood pressure, mainly diastolic, which is thought to be related to the drug's direct effect on NO as a scavenger and direct inhibition of eNOS (Figure 3) [36,37]. Other identified mechanisms include directly inhibiting guanylate synthase [1], its ability to bind hydrogen sulfide (H_2_S), a natural vasodilator produced by the endothelium, resulting in hypotension [14], and downstream modulation of cytokine pathways [38]. 

The dosage of hydroxocobalamin for the treatment of vasoplegia is determined by its primary indication of acute cyanide toxicity, which is a 5-10 g IV infusion over 15 minutes. Hydroxocobalamin is generally a well-tolerated drug. However, it's not free of side effects. The unfavorable consequences are mainly caused by hypersensitivity reactions related to cobalt or other injection-related substances. Transient chromaturia (red or blue discoloration of urine after administration that resolves spontaneously), exanthema (rash), itching, fever, nausea, dizziness, rigors, and hot flashes are common side effects [39]. Other less commonly reported side effects are transient hypokalemia in patients with megaloblastic anemia [40], and false activation of the blood leak alarm in the hemodialysis machine [41]. On rare occasions, hydroxocobalamin has also led to the development of severe anaphylactic reactions [42]. Despite having an overall safer profile when compared with methylene blue, high doses of hydroxocobalamin are significantly more expensive and may not be available in all hospital stocks, especially with its limited indications.

Hemoperfusion and hemodialysis are ineffective in treating CCB toxicity given their inability to clear the protein-bound CCB molecules [43] and due to their volume of distribution [44]. However, patients with CCB overdose may be good candidates for MARS therapy. MARS is an extracorporeal liver support system combining filtration, adsorption, and dialysis processes in its mechanism of action. It entails the removal of toxins from the blood, including albumin-bound and water-soluble compounds, aiding in the elimination of toxic metabolites accumulated in cases of hepatic failure [45].

A few case reports have demonstrated the effective use of MARS therapy as a last resort when all other measures fail [27,46]. Beyls C et al. reported the largest case series of MARS therapy for CCB poisoning on seven patients and observed its therapeutic effect without the development of serious adverse effects [47]. The efficacy and safety of MARS for treating CCB poisoning should be further investigated in controlled clinical trials, which would optimize evidence-based decision-making in treating patients with a CCB overdose.

## Conclusions

Vasoplegia is a type of distributive shock that is characterized by a significant reduction of SVR due to profound vasodilation. This causes severe hypotension in the context of normal or increased cardiac output, which is nonresponsive to adequate fluid resuscitation. The mainstay treatment modalities for vasoplegia include volume resuscitation, catecholamines, vasopressin, and angiotensin II. However, in circumstances where these modalities fail, other agents, such as methylene blue and hydroxocobalamin, can be employed due to their inhibitory actions on NO. In our patient, the administration of high-dose insulin, as well as the use of hydroxocobalamin, resulted in significant clinical improvement by resolving vasoplegia, increasing blood pressure, and restoring tissue perfusion. Currently, there are no published randomized clinical trials to assess the utility of hydroxocobalamin in the treatment of vasoplegia. This case report could serve as evidence to support the implementation of such studies.
